# Correlation of Systolic and Diastolic Blood Pressure in Measuring Blood Pressure Using Validated Digital Wrist Monitor Versus Validated Digital Arm Monitor: Study in a Real‐World Setting

**DOI:** 10.1155/ijhy/6941796

**Published:** 2026-05-07

**Authors:** Arnold Benjamin Mina, Marlon Co, Alejandro F. Diaz, Benjamin A. Balmores, Dolores Bonzon, Leilani B. Mercado-Asis

**Affiliations:** ^1^ Section of Cardiology, Department of Internal Medicine, Adventist University of the Philippines - College of Medicine, Silang, 4118, Cavite, Philippines; ^2^ Section of Cardiology, Department of Internal Medicine, Cebu Doctors University Hospital, Cebu, 6000, Philippines; ^3^ Department of Neuroscience and Behavioral Medicine, Neuroscience Institute, University of Santo Tomas Faculty of Medicine and Surgery, Sampaloc, 1008, Manila, Philippines; ^4^ Section of Nephrology, Department of Internal Medicine, St. Luke’s Medical Center, Quezon, 1112, Philippines, stluke.com.ph; ^5^ Division of Pediatric Nephrology, University of the Philippines-Philippine General Hospital, Ermita, 1000, Manila, Philippines, pgh.gov.ph; ^6^ Section of Endocrinology, Diabetes and Metabolism, Department of Medicine, University of Santo Tomas Faculty of Medicine and Surgery, Sampaloc, 1008, Manila, Philippines

**Keywords:** blood pressure correlation, measuring blood pressure, real-world setting, systolic and diastolic blood pressure, wrist BP versus arm BP

## Abstract

Wrist‐cuff blood pressure (wBP) monitors in the market have gained popularity due to their cost, portability, acceptability, and accessibility. Although wide variability in wrist versus the standard arm BP (aBP) monitor values has been reported, comparative investigations in a real‐world setting has not been fully elucidated. This study was undertaken to correlate systolic and diastolic BP using wBP versus the standard aBP monitors, to determine discrepancies, if any, and to formulate recommendations for its use. Adult subjects with normal, mild, or moderate hypertension randomly recruited in the investigators’ clinics were included. Complete medical history and physical examination were done, and standard positioning for BP measurements was followed. Validated wrist BP (Omron, HEM6161) and validated aBP (Omron, HEM7156) monitors were used. The statistical data were analyzed with *R* (ver 4.3.2) and MedCalc Statistical Software (Version 22.021). Correlation between wBP and aBP was determined using Passing‐Bablok regression and Lin’s concordance correlation coefficient. Bland–Altman analysis was applied to determine if the wBP values were according to the predetermined clinical significance for systolic and diastolic BP. Statistical significance was defined as a *p* value of less than 0.05 for all tests. Two‐hundred ninety‐nine (299) patients participated in the study, with a mean (SD) age of 44.2 years ± 15.1. Majority of the hypertensive subjects were on medications. There were 45 (15%) participants with aBP in the hypertensive level (BP > 140 mm Hg and diastolic BP > of 90 mm Hg). The median systolic wBP was 127 mmHg (range, 115–138) while aBP was 129 mmHg (range, 116–140). The median diastolic wBP and aBP were 80 mmHg (range, 74–88) and 82 mmHg (range, 75–88), respectively. A statistically significant correlation (*p* = 0.002 and *p* = 0.003) was obtained between the systolic and diastolic measurements using wBP and aBP apps. However, diastolic wBP was systematically lower by 10 mmHg. In conclusion, there is a correlation between the systolic and diastolic BP values taken through a validated digital wrist BP monitor and a validated aBP monitor. However, caution must be observed when interpreting the diastolic BP results. Clinical correlation is imperative.

## 1. Introduction

Hypertension remains to be the number one risk factor for heart disease and stroke, and yet the number of hypertensives with controlled blood pressure (BP) remains low. The World Health Organization (WHO) estimates worldwide prevalence rates around 30%, with almost 60% living in low to middle income countries (LMICs). Less than half are diagnosed and treated, and only 1 out of 5 has controlled BP [1]. In the Philippines, which falls under LMIC, it remains to be an important cause of morbidity and mortality and poor BP control has been identified to be one of the main causes [2].

In the most recent report from the Philippine Society of Hypertension (PSH), citing 2021 results from the May Measurement Month BP screening campaign, hypertension awareness in the country is only 54% and BP control is achieved in less than half of the treated patients. The need for improved BP screening programs in the country has been emphasized [3].

In a country such as the Philippines, the socioeconomic impact of uncontrolled hypertension is enormous and the burden of care can be challenging. This was shown in the projection study done by the PSH, calculating the cost of illness and its impact on productivity at work and at home, including loss of productivity due to premature mortality. The economic cost of hypertension was projected to increase from US$1 billion in 2020 to US$1.9 billion by the year 2050 [4]. This amount can be overwhelming in a LMIC such as the Philippines, hence the need for better BP control through monitoring and improved awareness.

Most experts recommend home blood pressure monitoring (HBPM) to improve adherence to medications and thereby control. The use of digital BP monitors has enabled patients to do self‐monitoring of the blood pressure (SMBP) at home, which leads to improved awareness and improved adherence leading to attainment of target BPs in majority of patients [5]. Compared with ambulatory blood pressure monitoring (ABPM), HBPM is cheaper, repeatable, more convenient, and easier to perform. However, these devices require validation [6].

Even though hypertension guidelines recommend the use of arm BP (aBP) monitors in the office or clinic for the diagnosis of hypertension and out‐of‐office monitoring at home [2], wrist BP monitors have gained popularity in the past due to their ease of use, lower cost, portability, patient acceptance, and easy access through online purchases [7]. Nonvalidated BP monitors are more widely used, especially coming from online sources because of its lower cost. If patients use nonvalidated monitors that are not proven accurate according to standards, BP readings might be inaccurate, resulting in under‐ or overdiagnosis, and this may affect subsequent treatment [8]. This has raised enormous concern due to wide variability of BP values between the aBP and wrist BP monitors, which could significantly alter the doctor’s clinical decision specifically on adjustment of medications [9–11]. Nonetheless, the use of wrist BP monitors continues to increase, especially in the frail, elderly, and obese individuals [12–14].

Newer models of wrist BP monitors have updated algorithms with the addition of position sensors to improve accuracy [15]. Currently, to this date, there are no new studies comparing the BP values taken with an aBP monitor simultaneously to that of a wrist BP monitor done in a real‐world setting, hence the investigators deemed it necessary to come up with this comparative study. In this study, we aim to correlate and compare BP measurements taken via wrist BP and aBP monitors, determine if there is any discrepancy in the readings, and recommend the specific utilization of the wrist BP monitor in the general population.

## 2. Methodology

Adult subjects between 18 and 80 years old with either normal or mild to moderate hypertension were recruited in the investigators’ clinics. Hypertension was defined as systolic BP = / > 140 and/or diastolic BP = / > 90 mmHg. Patient evaluation was performed, including a complete history and medication use and physical examination. Comorbidities were asked, especially pertaining to the exclusion criteria, which include severe hypertension (systolic BP 220 and above and/or diastolic BP 120 and above), cardiac arrhythmia (atrial fibrillation), post stroke patients, post myocardial infarction patients, steroid therapy for the past 3 months before the study, chronic kidney disease stages 4 and 5, cancer, psychiatric patients, pregnant patients, and movement disorders.

Subjects were instructed to rest quietly in the waiting room while waiting for their turn and were told to avoid smoking, intake of caffeine, and exercising for at least 30 min prior to BP measurement. Subjects were then asked to sit down and place their arm on the table for support when using the aBP monitor, or asked to sit down, place their arm on the table with elbows bent to place their wrist (hand with open palms) level with the heart when using the wrist monitor. The standard position recommended by the Philippine CPG on hypertension will be used for arm measurements, while the recommended arm and wrist position will be followed using the wrist BP monitor instruction leaflet.

The investigators used validated arm and wrist BP monitors that were widely available in the country. For the aBP monitor, Omron HEM 7156 was used while for the wrist BP monitor, Omron HEM 6161 was used. Validation studies for the said monitors or their equivalent have been published online and available for retrieval [16–18]. Equivalence certificates are likewise available from the company by request.

The BP will be alternately measured, first using the arm monitor, followed within 1 min by the wrist monitor. There will only be 1 measurement for each monitor per participant, for a total of 2 BP measurements per participant. There was a total of six investigators recruiting around 50 subjects each, multiplied by 2 measurements per participant, yielding a total of around 600 BP measurements available for analysis for this study, yielding a reasonable sample size in method comparison studies [19].

### 2.1. Statistical Analysis

The statistical data were analyzed with *R* (ver 4.3.2) and MedCalc Statistical Software (Version 22.021). Passing–Bablok regression was used to measure the agreement, which includes the systematic and proportional bias of the aBP and wrist BP measurements. Bland–Altman analysis was applied to determine if the wrist measurements were according to the predetermined clinical significance for systolic and diastolic BP. Correlation analysis was also conducted using Lin’s concordance correlation coefficient (*r*_*c* *c* *c*_). Furthermore, the recommended wrist diastolic BP adjustment based on the Passing–Bablok regression calculated the changes in the sensitivity and specificity of hypertension diagnosis. Statistical significance was defined as a *p* value of less than 0.05 for all tests.

## 3. Results

### 3.1. Demographics of Subjects

Table 1 shows two‐hundred ninety‐nine (299) patients who participated in the study, with a mean age of 44,2 years (range, 18–82) and a mean BMI of 25.8 kg/m2 (range, 15.5–46.1). A total of 141 (47.2%) patients have normal BMI, while 7 (2.3%) are underweight, 98 (32.8%) are overweight, and 53 (17.7%) are obese. Most of the participants are female (*n* = 175, 58.5%). The majority of hypertensive subjects were on medications. There were 45 (15%) participants with aBP in the hypertensive level (systolic BP > 140 mm Hg and diastolic BP > 90). The median systolic BP using the aBP is 129 mm Hg (IQR, 116–140) while using the wrist BP is 127 mm Hg (IQR, 115–138). As for the diastolic BP, using the aBP is 82 mm Hg (IQR, 75–88) and 80 mm Hg (IQR, 74–88) using the wrist BP.

**TABLE 1:** Profile of participants.

Demographic	Summary^∗^
Age (years)	44.2 ± 15.1
Sex: Male	124 (41.5%)
Female	175 (58.5%)
BMI (Kg/m^2^): act	25.8 ± 5.0
BMI class:	
Obese (at least 30 kg/m^2^)	53 (17.7%)
Overweight (25–30 kg/m^2^)	98 (32.8%)
Normal (18.5–25 kg/m^2^)	141 (47.2%)
Underweight (below 18.5 kg/m^2^)	7 (2.3%)

^∗^Values expressed as mean ± SD, or *n* (%).

### 3.2. Comparison of Wrist BP Versus aBP

Figures 1 and 2 show the histogram of the systolic and diastolic pressure measurements using the wrist BP and aBP. As shown in Table 2, the median of the systolic BP using the arm is 129 mm Hg (IQR, 116–140) while using the wrist is 127 mm Hg (IQR, 115–138). As for the diastolic BP, using the arm is 82 mm Hg (IQR, 75–88) and 80 mm Hg (IQR, 74–88) using the wrist.

FIGURE 1(a) Arm and (b) wrist systolic blood pressure (mm Hg) of the patients.

**Figure:**
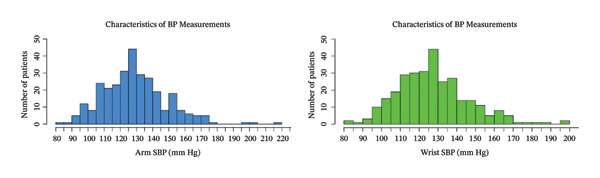
(a)

**Figure:**
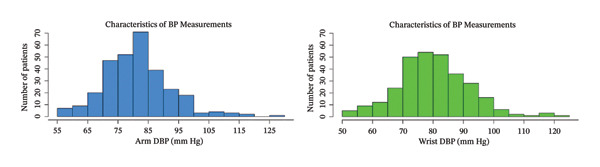
(b)

FIGURE 2(a) Arm and (b) wrist diastolic blood pressure (mm Hg) of the patients.

**Figure:**
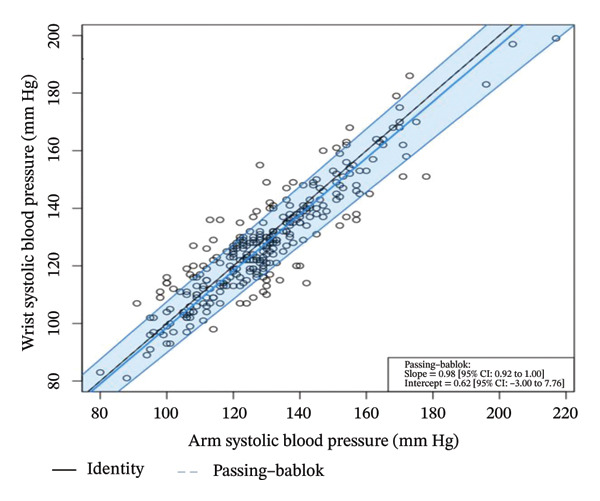
(a)

**Figure:**
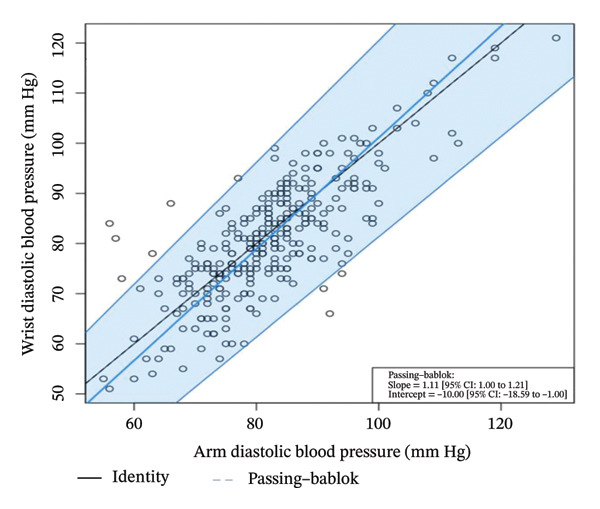
(b)

**TABLE 2:** The distribution of arm and wrist systolic and diastolic blood pressure.

	**Mean**	**SD**	**Min**	**25** ^**th**^ **percentile**	**50** ^**th**^ **percentile**	**75% percentile**	**Max**

Systolic BP (mm hg)							
Arm	129.41	19.90	80	116	129	140	217
Wrist	127.87	19.07	81	115	127	138	199
Diastolic BP (mm Hg)							
Arm	82.05	11.05	55	75	82	88	129
Wrist	80.76	11.72	51	74	80	88	121

With the Passing–Bablok regressions, in measuring the systolic BP, there is no systematic (Intercept = 0.62 (95% confidence interval [CI]: −3.00–7.76)) and no proportional differences (Slope = 0.98 (95% CI: 0.92–1.00)) in the arm and wrist measurements. However, although there were no proportional differences (Slope = 1.11 (95% CI: 1.00–1.21)) in measuring the diastolic BP, it was evident that there is a systematic difference (or constant bias) (Intercept = −10.00 (95% CI: −18.59 to −1.00)) in the diastolic BP measurements using the wrist. This shows that the diastolic BP measurements using the wrist are, on average, lower than 10 mm as compared with the diastolic BP measurements using the arm. Figure 3 presents the Passing–Bablok regressions, with 95% CI across the systolic and diastolic BP readings.

FIGURE 3Bland–Altman plots of the arm and wrist systolic (a) and diastolic (b) blood pressure measurements.

**Figure:**
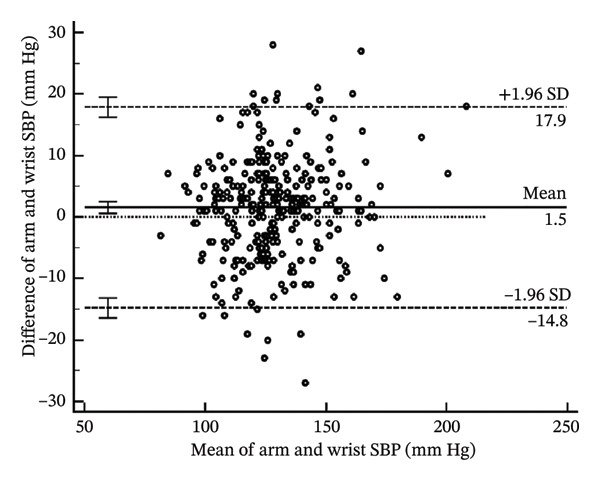
(a)

**Figure:**
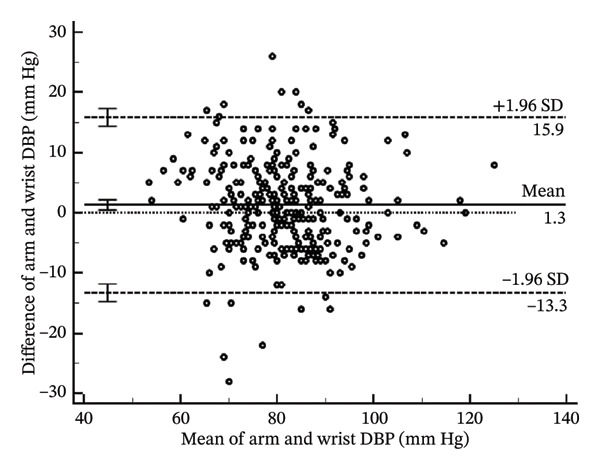
(b)

Table 3 shows that in measuring the systolic BP, there is no systematic (Intercept = 0.62 (95% CI: −3.00–7.76)) and no proportional differences (Slope = 0.98 (95% CI: 0.92–1.00)) in the arm and wrist measurements. However, although there was no proportional differences (Slope = 1.11 (95% CI: 1.00–1.21)) in measuring the diastolic BP, it was evident that there is a systematic difference (or constant bias) (Intercept = −10.00 (95% CI: −18.59 to −1.00)) in the diastolic BP measurements using the wrist. This shows that the diastolic BP measurements using the wrist are, at an average, lower than 10 mm as compared with the diastolic BP measurements using the arm. The CUSUM test for linearity indicates that the Passing–Bablok regression analysis has been applicable in the analysis of systolic (*p* = 0.770) and diastolic (*p* = 0.480) BP measurements using the arm and wrist.

**TABLE 3:** Passing–Bablok analysis.

	**Intercept**	**Slope**	**RSD**	**CUSUM**

Systolic BP (mm Hg)	0.62 (−3.00–7.76)	0.98 (0.92–1.00)	5.88 (−11.53 to 11.53)	0.77
Diastolic BP (mm Hg)	−10.00 (−18.59 to −1.00)	1.11 (1.00–1.21)	5.25 (−10.30 to 10.30)	0.48

*Note:* Values in parentheses are the 95% confidence intervals; RSD: residual standard deviations; CUSUM: the *p* value of the CUSUM test for linearity. An intercept with 95% CI including 0 indicates no constant bias and a slope with 95% CI including 1 indicates no proportional bias.

To help determine systematic error and whether it is uniform across the range of results, Bland–Altman analysis (Figure 4) determines the differences when arm systolic and diastolic BP measurements were subtracted from the wrist measurements. The mean bias for the systolic BP was 1.54 mm Hg (95% CI: 0.59–2.49) (*p* = 0.002). The lower limit of agreement (LoA) was −14.78 mm Hg (95% CI: −16.40–−13.16), and the upper LoA was 17.86 mm Hg (95% CI: 16.23–19.48) (Table 4). The results show that systolic BP readings using the wrist may be as low as the arm to 17.86 mm Hg and higher than the arm to 14.78 mm Hg across the range. Thus, the systolic BP using the wrist measurement is acceptable since it is within the pre‐established acceptable LoA of 20 mm Hg.

FIGURE 4Bland–Altman plots of the arm and wrist (a) systolic and (b) diastolic BP measurements.

**Figure:**
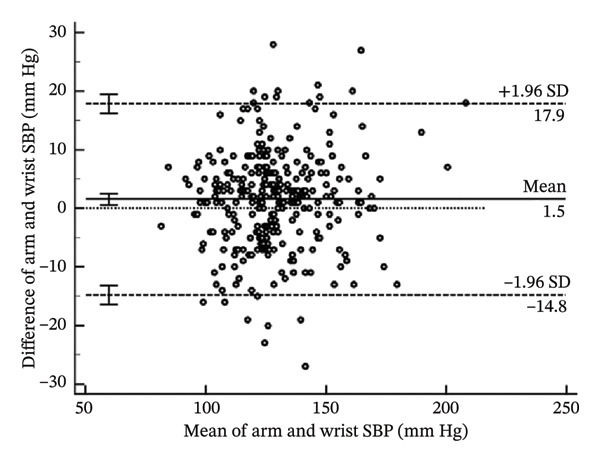
(a)

**Figure:**
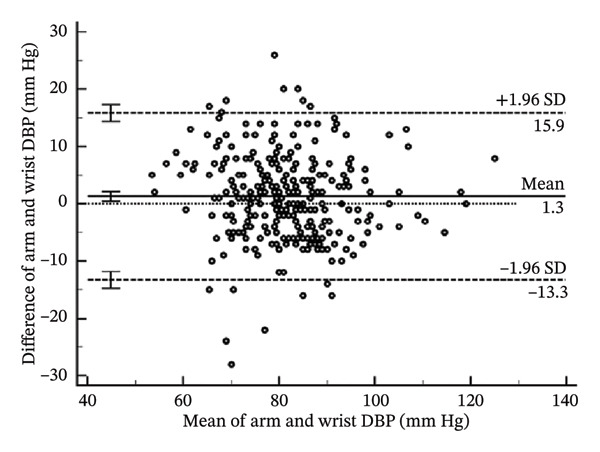
(b)

**TABLE 4:** Bland‐Altman analysis of the differences of arm BP–wrist BP.

	**Mean**	**Lower LoA**	**Upper LoA**

Systolic BP (mm Hg)	1.54 (0.59–2.49)	−14.78 (−16.40 to −13.16)	17.86 (16.23–19.48)
Diastolic BP (mm Hg)	1.29 (0.44–2.13)	−13.29 (−14.74 to −11.84)	15.87 (14.42–17.31)

*Note:* Values in parentheses are the 95% confidence intervals. LoA; lower limit of agreement.

As for the diastolic BP, the mean bias was 1.29 mm Hg (95% CI: 0.44–2.13) (*p* = 0.003). The lower LoA was −13.29 mm Hg (95% CI: −14.74 to −11.84), and the upper LoA was 15.87 mm Hg (95% CI: 14.42–17.31). This shows that diastolic BP readings using the wrist may be as lower than the arm to as much as 15.87 mm Hg and higher than the arm to as much as 13.29 mm Hg across the range. Thus, the diastolic BP using the wrist measurement is unacceptable since it is not within the pre‐established acceptable LoA of 10 mm Hg.

### 3.3. Concordance Analysis

A concordance analysis (Table 5) indicates moderate agreement in arm and wrist measurements of the systolic BPs (*r*_*c* *c* *c*_ = 0.906 (95% CI: 0.884–0.924); *ρ* = 0.910; and *C*_*b*_ = 0.996) but poor agreement for the diastolic BPs (*r*_*c* *c* *c*_ = 0.782 (95% CI: 0.734–0.822); *ρ* = 0.788; and *C*_*b*_ = 0.992).

**TABLE 5:** Lin’s concordance analysis.

	**Lin’s** **r**_**c** **c** **c**_	**Precision (** **ρ** **)**	**Accuracy (** **C**_**b**_ **)**

Systolic BP (mm Hg)	0.906 (0.884–0.924)	0.910	0.996
Diastolic BP (mm Hg)	0.782 (0.734–0.822)	0.788	0.992

*Note:* Values in parentheses are the 95% confidence interval. An *r*_*c* *c* *c*_ value of < 0.90 was considered poor agreement, 0.90–0.95 moderate agreement, 0.95–0.99 substantial agreement, and ≥ 0.99 near perfect agreement.

Since the *r*_*c* *c* *c*_ is the product of the precision *ρ* and accuracy (*C*_*p*_), it shows that despite high accuracy (*C*_*b*_ = 0.992) of diastolic BP, the poor agreement (*r*_*c* *c* *c*_ = 0.782) is due to precision’s low value (*ρ* = 0.788).

## 4. Discussion

### 4.1. Clinical Usefulness of Wrist BP

There is a need to monitor out‐of‐office BP to improve awareness and control of hypertension, but it is imperative to use clinically validated monitors to ensure accurate and reliable measurements. It is also important that these monitors should be easy to use for self‐monitoring, especially in the elderly population. The guidelines still recommend the use of aBP monitors for the diagnosis of hypertension and discourage the use of wrist BP monitors due to concerns on accuracy. However, for out‐of‐office BP monitoring/HBPM/SMBP, the wrist monitor is still a promising alternative, especially the newer updated monitors using position sensors to improve accuracy.

Wrist monitors are appealing because of its small size, making it more portable and easy to use. It has a wide range of use, for example, obese individuals with shorter arm length to arm circumference (conical‐shaped arms), elderly who might find it physically challenging to apply the arm cuffs properly, post breast surgery patients, travelers who desire a more portable monitor, and young individuals who find smaller monitors to be more acceptable to use [10].

However, with regard to the use of wrist BP in obese individuals, it should be noted that wrist BP cuffs have a limit of 13.5–21.5 cm wrist diameter, hence for severely obese individuals with wrist diameter > 21.5 cm, it will not be useful. In addition, these individuals may also have conical wrists as well, such that for severely obese individuals with wrist circumference > 21.5 cm and conical wrists, the best monitor to use will still be the aBP monitor due to availability of special “tronco‐conical” aBP cuffs. The usual “cylindrical” cuffs, even if large sizes are used in severely obese individuals with conical arms, has been shown to overestimate the BP, especially in those with baseline elevated BP. The use of a “tronco‐conical” cuff is recommended in these individuals for improved accuracy [20].

### 4.2. Addressing Wrist BP and aBP Measurement Values

Previous comparisons between aBP and wrist BP measurements yielded variable results. The wrist BP monitor’s shortcoming in approximating the aBP monitor’s measurements has been identified to be due to several factors, namely, positioning of the arm [21], rigorous activity of the person [22], and administering specific medications such as anesthesia [23]. Notably, Komori and colleagues showed fair agreement comparing a wrist monitor with the arm monitor in the ambulatory condition [24]. Furthermore, with the use of a position sensor, the wrist‐cuff BP (wBP) monitor passed the International Protocol criteria to be used in adults and obese individuals [13]. In a recent report by Kario et al, they demonstrated that wBP correlated well with aBP and provided ease in monitoring nighttime BP, substantially reducing sleep disturbance compared with the arm monitor, thus providing convenience for both the patient and the healthcare provider [25].

In early studies comparing BP values taken with a wrist BP monitor to values taken using the arm via mercury sphygmomanometer and 24‐h ABPM, the wrist monitor yielded results comparable to the two other methods. The authors then recommended that wrist BP monitors may be used in place of ABPM, giving a more practical and repeatable method in monitoring of the BP at home. Need for further comparative studies was recommended [26].

### 4.3. Use of Single BP Measurement per Device per Person

Although some protocols recommend 3 BP measurements when comparing 2 methods of measurement, the authors of this study performed only 1 measurement for each device per participant, and this might be viewed as a limitation of this study. However, the statistical method used for analysis is Passing–Bablok regression, which is intended for method comparison using paired single measurements per participant. Using only one value avoids correlated replicates and overestimation of precision, and using one measurement per participant per method maintains the independence of observations [27]. To avoid wide variability between aBP and wrist BP readings, the investigators measured the aBP and wrist BP within 1 min.

### 4.4. Use of Wrist BP Measurement in the Real World: Significance of Our Study

In our study, using updated, validated wrist BP monitors in comparison to validated aBP monitors, the measurement of the systolic BP showed no difference between the aBP and the wrist BP monitors. However, for the diastolic blood pressure, measurements using the wrist monitor showed a lower measurement of about 10 mmHg compared with the arm monitor, on the average.

Further analysis showed that while systolic BP readings using the wrist monitor may be acceptable compared with the arm monitor (since it is within the pre‐established acceptable LoA within 20 mmHg), measurements for the diastolic BP may not be acceptable using the wrist monitors (since it is not within the pre‐established acceptable LoA within 10 mmHg). These LoAs were reported by 2 authors, wherein Stergiou et al. noted that differences of up to 10–15 mm Hg between BP readings are common even under standardized conditions, while Beevers et al. reported that discrepancies may reach up to 20 mm Hg for systolic BP and 10 mm Hg for diastolic BP [28, 29].

Passing–Bablok regression shows that the wrist measurements underestimates the arm diastolic measurements by 10.0 mm Hg such that to improve the diastolic BP measurements, it is recommended to add 10.0 mm Hg for adjustment of the wrist diastolic BP. When the wrist diastolic BP was adjusted by adding 10.0 mm Hg, the sensitivity of wrist measurements in identifying hypertensive patients went higher to 77.8% (95% CI: 62.9%–88.8%) and its specificity went lower to 90.2% (95% CI: 85.8%–93.5%). The overall accuracy was reduced to 88.3% (95% CI: 84.1%–91.7%).

It was previously reported that arm position and pulse pressure has an impact on wrist BP measurement, comparing three positions: “horizontal” (wrist at heart level), “shoulder” (hand supported on the opposite shoulder), and “desk” (elbow placed on desk). Results showed sBP readings to be closest to aBP readings with the wrist BP taken in the “desk” position. An adjustment of 5 and 10 mmHg for sBP and dBP (respectively) in the “desk” position resulted in improved readings. A mismatch between aBP and wrist BP was said to be directly associated with pulse pressure [26]. Hence, proper training for the use of wBP monitors should be emphasized because wBP measurements are dependent on wrist–forearm angle and wrist–heart hydrostatic height difference [10].

In our study, we used the latest design wrist BP monitor from Omron using updated algorithms, with wrist position sensors designed to be used in the “desk” position, which was found to closely correlate with aBP.

## 5. Conclusion

Monitoring BP to improve awareness and control of hypertension using validated digital monitors ensures accurate and reliable measurements. These monitors should be easy to use for self‐monitoring. Wrist BP monitors hold promise for out‐of‐office monitoring, especially newer wrist BP monitors that use position sensors to improve accuracy. Overall, there is a correlation between the systolic and diastolic BP values taken through a validated digital arm and validated digital wrist BP monitor. Care must be taken to ensure proper positioning of the wrist and hand since wrist monitors are sensitive to positioning. Validated wrist monitors are to be used only for out‐of‐office monitoring since current guidelines recommend the use of aBP monitors for the “diagnosis” of hypertension. A validated wrist monitor can provide accurate measurements for out‐of‐office monitoring if used as directed, but it should be periodically counterchecked in the office by the healthcare provider. Still, caution must be observed when interpreting the diastolic BP results. This study has shown that systolic BP values taken by wrist were acceptable. However, there was a 10 mm Hg discrepancy observed in the diastolic BP when comparing arm and wrist. An adjustment of plus 10 mmHg for the dBP is recommended. Clinical correlation is imperative.

## Funding

This study is author‐initiated, but Omron Philippines provided the BP monitors and financial support in the form of allowance for the participants of the study and funding for publication.

## Conflicts of Interest

The authors declare no conflicts of interest.

## Data Availability

The data that support the findings of this study are available on request from the corresponding author. The data are not publicly available due to privacy or ethical restrictions.
